# Genetic variant in miR-17-92 cluster binding sites is associated with esophageal squamous cell carcinoma risk in Chinese population

**DOI:** 10.1186/s12885-022-10360-6

**Published:** 2022-12-02

**Authors:** Yi Shen, Yi Shao, Xiaoli Ruan, Lingyan Zhu, Zhaoping Zang, Tong Wei, Rena Nakyeyune, Wenqiang Wei, Fen Liu

**Affiliations:** 1 Department of Epidemiology and Health Statistics, School of Public Health, Beijing Municipal Key Laboratory of Clinical Epidemiology, Capital Medical University, Beijing, 100069 China; 2 National Central Cancer Registry, National Cancer Center/National Clinical Research Center for Cancer/Cancer Hospital, Chinese Academy of Medical Sciences and Peking Union Medical College, Beijing, 100021 China

**Keywords:** Esophageal squamous cell carcinoma, miR-17-92 cluster, Single nucleotide polymorphism, Binding sites, Genetic susceptibility

## Abstract

**Background:**

Single nucleotide polymorphisms (SNPs) located in microRNA (miRNA) binding sites can affect the interactions between miRNAs and target genes, which is related to cancer susceptibility and tumorigenesis. However, the association between SNPs located in miR-17-92 cluster binding sites and ESCC risk remains unclear. Therefore, we aimed to explore the relationship between polymorphisms in miR-17-92 cluster binding sites and ESCC susceptibility.

**Methods:**

Six SNPs in the binding sites of miR-17-92 cluster were selected using bioinformatics databases, and their association with ESCC risk was investigated in a case-control study (including 488 cases and 512 controls) based on the population from high incidence areas of ESCC in China. We evaluated the SNP-SNP and SNP-smoking interactions using generalized multifactor dimensionality reduction (GMDR). Moreover, the expression of the miR-17-92 cluster and its target genes was determined in ESCC and adjacent normal tissues by quantitative real-time polymerase chain reaction (qRT-PCR). The dual-luciferase reporter assay was conducted to verify the effect of SNPs on the binding affinity between miRNAs and target genes.

**Results:**

We found that the SNP rs1804506 C > T had a significant association with the decreased ESCC risk. The SNP rs1804506 T allele was associated with a significantly decreased risk of ESCC in the additive model (OR = 0.817, 95% CI = 0.681–0.981, *P* = 0.030). The rs1804506 T allele had more striking effects on reducing ESCC risk in older individuals, female or non-smoker subgroups. We also found a significant interaction effect between rs1366600 and smoking by GMDR methods (*P* = 0.011). Additionally, the expression levels of miR-19a-3p and TGFBR3 were significantly downregulated in ESCC tissues compared with normal tissues, and the carriers of rs1804506 TT genotype had lower expression level of TGFBR3 than those of rs1804506 CC/CT genotype. Following dual-luciferase reporter assay showed that the rs1804506 T allele reduced the binding of miR-19a-3p and TGFBR3 3′-UTR.

**Conclusions:**

Our findings suggest that the rs1804506 polymorphism in miR-17-92 cluster binding sites contributes to the susceptibility of ESCC, which might provide new clues and scientific evidence for the etiology and biomarkers for the prevention and treatment of ESCC.

**Supplementary Information:**

The online version contains supplementary material available at 10.1186/s12885-022-10360-6.

## Background

Esophageal cancer (EC) is one of the most common gastrointestinal cancers. It is estimated that there are 604,100 new esophageal cancer cases and 544,076 deaths in 2020 worldwide, which ranks seventh in incidence and sixth in mortality of all malignancies [1]. Esophageal squamous cell carcinoma (ESCC) is the most common and predominant histologic subtype, with a proportion of nearly 90% in China [2]. China is one of the areas with the highest incidence rates of EC, accounting for approximately 50% of all cases in the world [1]. Despite the descending incidences in last few decades, ESCC remains a public health concern in China, with a 5-year survival rate of 30% [3]. ESCC is considered as a complex disease closely related to the environment and genetic factors as well as their interactions. Notably, identification of candidate biomarkers or genetic risk factors would greatly improve the survival rates of ESCC [4]. Although considerable efforts have been devoted to exploring the carcinogenesis of ESCC, its genetic pathogenesis remains unclear. Therefore, it is necessary to identify valuable markers for the surveillance, prognosis and therapeutic intervention of ESCC.

MicroRNAs (miRNAs), a class of endogenous, small non-coding RNA molecules constituting of 18 ~ 25 nucleotides, could bind to the 3′-untranslated regions (3′-UTRs) of target messenger RNAs (mRNAs), leading to the degradation or translation inhibition of mRNAs, thus negatively regulating gene expression at the post-transcriptional level [5]. MiRNAs are involved in a variety of important biological processes including cell proliferation, differentiation, and apoptosis [5, 6]. More and more studies have revealed that some miRNAs play important roles in the development of malignant tumors. Existing evidence has indicated that miRNAs function as oncogenes or tumor-suppressors in the tumorigenesis of ESCC [7, 8], and our previous researches suggest that circulating miRNAs have the potential as candidate biomarkers for ESCC [9, 10]. Furthermore, it is notable that some miRNA-related single nucleotide polymorphisms (miR-SNPs), for example, miR-SNPs rs6505162 [11], rs2910164 [12], and rs11614913 [13] were closely related to the susceptibility of ESCC and played important roles in tumorigenesis. Our former study revealed that miR-SNP rs1451761 was significantly associated with ESCC risk, which might serve as a functional susceptibility locus in the development of ESCC [14]. Moreover, miR-SNPs located in 3′-UTRs of target genes can alter the binding ability between miRNAs and target genes, which affect the post-transcriptional regulation and might serve as promising biomarkers [15, 16]. Up to now, miRNASNP-v3 database has provided more than seven million SNPs located in the 3’UTRs that might affect miRNA target binding [17]. Polymorphism rs11473 C > T at the 3′-UTR of BSG was significantly associated with a high risk of esophageal cancer [18], whereas polymorphism rs373245753 T > G at the CAPZA1 3′-UTR could disrupt the binding of miR-875-5p and the target CAPZA1 [19]. The rs6573 A > C, located in the RAP1A 3′-UTR, contributed to ESCC susceptibility and interfered the binding of miR-196a and RAP1A [20].

The miR-17-92 cluster (including miR-17, miR-18a, miR-19a, miR-20a, miR-19b-1, and miR-92a-1), encoded by the miR-17-92 host gene, is considered as the most characteristic polycistron and contributes to the process of tumor pathogenesis. Recent studies have reported that the miR-17-92 cluster was differentially expressed in various tumors including liver cancer, lung cancer, colorectal cancer and B-cell lymphoma [21–23]. In addition, the miR-17-92 cluster was observed to have abnormal expression patterns in ESCC tissues, which could regulate the target gene expression and have promotional effects on the proliferation, migration, and invasion in ESCC cells [24]. Our previous research has indicated that miR-92a-3p, a member of the miR-17-92 cluster, was aberrantly increased in the serum of ESCC and esophageal squamous dysplasia patients, and it showed a good performance for the detection of ESCC and ESD patients [9]. However, the relationship between SNPs in the binding sites of miR-17-92 cluster and ESCC risk and their underlying mechanism is rarely investigated.

Therefore, we conducted a case-control study based on the population from high incidence areas of ESCC in China to investigate the association of miR-17-92 cluster polymorphisms and its altered expression with ESCC susceptibility. Additionally, dual-luciferase reporter assay was performed for a preliminary exploration of the mechanism of  the positive loci, which might provide new clues and scientific evidence for the prevention and treatment of ESCC.

## Methods

### Study population

We conducted a case-control study including 488 ESCC patients and 512 healthy controls. In brief, the ESCC patients were consecutively recruited from the Endoscopy Center of Cancer Hospital, Linzhou city, Henan province from 2014 to 2018. All patients were newly diagnosed and were histopathologically confirmed as ESCC without previous chemotherapy or radiotherapy. Healthy controls were randomly selected from individuals who participated in a community-based early EC screening program in Henan province during the same period. Information on age, gender and smoking history (current/former or never) was obtained by in-person interviews, which has been described in detail in our previous studies [9, 14]. This study was performed under the approval from the ethics committee of Capital Medical University (approval no. 2014SY31), and all procedures followed the principle of the Declaration of Helsinki. The written informed consent was obtained from each participant enrolled in this study.

### SNPs selection

Polymorphisms in miR-17-92 cluster binding sites were selected using bioinformatics databases: miRNASNP (http://bioinfo.life.hust.edu.cn/miRNASNP2/), mirsnpscore (http://www.bigr.medisin.ntnu.no/mirsnpscore/), PolymiRTS (http://compbio.uthsc.edu/miRSNP/), and MirSNP (http://bioinfo.bjmu.edu.cn/mirsnp/search/). In the miRNASNP database, putative miRNA target binding sites were predicted by TargetScan and miRmap tools [17]. The mirsnpscore online tool predicted the effects of SNPs in miRNA binding sites and mapped these miRNA-related variants to interested SNPs in GWAS using linkage disequilibrium [25]. The PolymiRTS database identified the predicted binding sites of interested SNPs and the miRNA seed regions using the TargetScan algorithm [26]. The MirSNP database provides human SNPs that located at miRNA-mRNA binding sites, which were mainly predicted by miRanda algorithm [27]. The candidate miR-SNPs and miRNA target binding sites were obtained by searching the miR-17-92 cluster in above databases. We also searched published literatures to screen cancer-related target genes. The HapMap CHB database was used to select SNPs according to the criteria of a minor allele frequency (MAF) > 5% in the Chinese Han population. Finally, six SNPs (rs12594531, rs1366600, rs1804506, rs3741779, rs3763763, and rs8323) were selected as candidate SNPs within the binding sites of miR-17-92 cluster. The selected miR-SNPs and the predicted binding sites between miRNAs and target genes were presented in Supplementary Fig. S1 (Additional file 1).

### DNA isolation and SNPs genotyping

Genomic DNA was isolated from the peripheral blood samples using the TIANamp Blood DNA Kit (Tiangen, Beijing, China). Site-specific PCR and detection primers were designed using MassARRAY Assay Design 3.0 software (Sequenom, San Diego, CA). The primer sequences were listed in Supplementary Table S1 (Additional file 2). The selected candidate SNPs were genotyped using the Sequenom MassARRAY platform (Sequenom, San Diego, CA) following the manufacturer’s instruction. The call rates for all SNPs were over 95%. Additionally, 5% of the samples were randomly selected to repeat genotyping for quality control, and the concordant rate for each SNP was 100%.

### RNA extraction and quantitative real-time PCR (qRT-PCR)

Fifteen pairs of ESCC tissues and adjacent normal tissues were collected from ESCC patients. The clinical characteristics are provided in Supplementary Table S2 (Additional file 2). Total RNA was extracted from tissue specimens using the TRI Reagent (Sigma) according to the manufacturer’s protocol. SuperScript™ III Reverse Transcriptase (Invitrogen) was used for reverse transcription. PCR assay with 2 × PCR master mix (Arraystar) was performed using a QuantStudio5 Real-time PCR System (Applied Biosystems). The reaction conditions were fulfilled by incubating at 95 °C for 10 minutes, and followed by 40 cycles of 95 °C for 10 seconds and 60 °C for 1 minute. The relative expression levels of miR-19a-3p, miR-19b-3p and TGFBR3 normalized to U6 and β-actin was calculated using the 2^−ΔΔCt^ method. All primer sequences for qRT-PCR were listed in Supplementary Table S1 (Additional file 2).

### Functional validation via expression quantitative trait loci (eQTL) databases

To explore the potential function of the studied SNPs, we examined its association with the expression of corresponding genes by querying eQTL databases. The Multiple Tissue Human Expression Resource (MuTHER) project performed eQTL analysis using the genotyping and gene expression data collected from 856 individuals enrolled in the UK Adult Twin Registry (http://www.muther.ac.uk/). Additionally, the eQTLGen Consortium (http://www.eqtlgen.org/), a huge-scale eQTL analysis containing 37 datasets with a total of 31,684 individuals, was used for functional validation [28]. We also investigated eQTL results from the Genotype-Tissue Expression (GTEx) database, which reported the association between SNP genotyping and gene expression levels in various human tissues [29].

### Plasmid construction and dual-luciferase reporter assays

The Human embryonic kidney 293 T (HEK-293 T) cells were obtained from Thermo Fisher Scientific and cultured in Dulbecco’s Modified Eagle’s Medium (DMEM, Thermo Scientific) containing 10% fetal bovine (Gibco) and grown in 5% CO_2_ incubator at 37 °C. The reporter plasmids containing the sequence of the TGFBR3 3′-UTR with rs1804506 C allele or T allele were constructed and cloned into the NotI/XhoI restriction enzyme sites of the psiCHECK-2 vector (Promega). DNA sequencing was used to verify all the cloned sequences. For luciferase assays, 2.0 × 10^4^ HEK-293 T cells in 100 μl growth medium were seeded in 96-well plates. Next, the has-miR-19a-3p mimics or its negative control (Oligobio) was co-transfected with constructed plasmids using Lipofectamine 2000 (Invitrogen). Forty-eight hours after transient transfection, the Dual Luciferase Reporter Assay System (Promega) was used to measure the luciferase activity according to the manufacturer’s instructions, and the Renilla luciferase activity was normalized to firefly luciferase activity. All assays were conducted in triplicate.

### Statistical analysis

The distribution of demographic characteristics in ESCC patients and healthy controls were compared by Student’s *t*-test or Pearson’s χ^2^ test. Hardy-Weinberg equilibrium (HWE) for the selected SNPs was evaluated by a goodness-of-fit χ^2^ test among the control group. Multivariable logistic regression analyses with adjustments for age, gender, smoking status and family history of cancers were employed to estimate the association of the selected SNPs with ESCC risk by computing adjusted odds ratios (ORs) and 95% confidence intervals (CIs). Generalized multifactor dimensionality reduction (GMDR) software was utilized to determine SNP-SNP and SNP-smoking interactions. We further assessed the association between the identified genetic interaction models and the ESCC risk by multivariable logistic regression analyses. Wilcoxon signed-rank test was used to compare the expression of miRNAs and their target genes among ESCC and adjacent normal tissues, and non-parametric analysis was used to compare miRNAs and target genes expression among individuals with different genotypes of miR-SNPs. Associations between miRNA and mRNA expression levels were determined using the Pearson’s correlation coefficient (r). Statistical Package for Social Science (SPSS) version 24.0 software was used for all statistical analyses unless otherwise specified. *P* values were two-sided, and the statistical significance was set at *P* < 0.05.

## Results

### Population characteristics

A population-based case-control study including 488 patients and 512 controls was performed to evaluate the association between the SNPs in miR-17-92 cluster binding sites and the risk of ESCC. The demographic characteristics of ESCC patients and healthy controls were shown in Table 1. The proportions of men (*P* = 0.002) and smokers (*P* < 0.001) in cases were higher than that in controls. No significant difference was detected in age (*P* = 0.682) and family history of cancers (*P* = 0.724) between ESCC and control groups.

**Table 1:** Demographic characteristics of ESCC patients and controls

Variables	Cases (%)	Controls (%)	*P*	OR (95%CI)
	*n* = 488	*n* = 512		
Age (mean ± SD), year	63.04 ± 7.09	62.89 ± 4.25	0.682^a^	
Gender				
Female	152 (31.1)	207 (40.4)	0.002^b^	1.000
Male	336 (68.9)	305 (59.6)		1.500 (1.156–1.947)
Smoking status				
Never	262 (53.7)	379 (74.0)	< 0.001^b^	1.000
Current/former	226 (46.3)	133 (26.0)		2.458 (1.884–3.207)
Family history of cancers				
No	278 (57.0)	286 (55.9)	0.724^b^	1.000
Yes	210 (43.0)	226 (44.1)		0.956 (0.744–1.228)

^a^*P*-value for Student’s *t*-test; ^b^*P*-value for a two-sided χ^2^ test; *CI* Confidence interval, *OR* odds ratio, *SD* Standard deviation

### Association between selected SNPs and ESCC susceptibility

Detailed information about the selected SNPs in miR-17-92 cluster binding sites was summarized in Table 2. Except for rs8323, the genotype frequencies of the other selected SNPs (rs12594531, rs1366600, rs1804506, rs3741779 and rs3763763) were in HWE among the control group (*P* > 0.05). Therefore, the above five SNPs were selected for further analysis. After adjusting for age, gender, smoking and family history of cancers, the SNP rs1804506 T allele was associated with a significantly decreased risk of ESCC in the additive model (OR = 0.817, 95% CI = 0.681–0.981, *P* = 0.030). A similar result was observed in the codominant model, where individuals carrying TT genotype had a decreased susceptibility for ESCC, compared with those carrying CC genotype (OR = 0.670, 95% CI = 0.463–0.968, *P* = 0.033). However, no statistically significant association was observed between the other miR-SNPs and ESCC risk (all *P* > 0.05) (Table 3).

**Table 2:** Detailed information of the selected SNPs

SNPs	Genes	Associated miRNAs	Location (GRCh 38)	Alleles^a^	Cases^b^ (*n* = 488)	Controls^b^ (*n* = 512)	MAF^c^	*P*_HWE_^d^
rs12594531	THSD4	miR-18a-5p	Chr15: 71782166	C/A	166/240/82	158/260/94	0.438	0.473
rs1366600	INSR	miR-17-5p/miR-20a-5p	Chr19: 7112870	A/G	353/122/13	347/149/16	0.177	0.999
rs1804506	TGFBR3	miR-19a-3p/miR-19b-3p	Chr1: 91682456	C/T	158/242/88	135/260/117	0.482	0.702
rs3741779	SSH1	miR-19a-3p/miR-19b-3p	Chr12: 108785032	C/T	232/221/35	238/219/55	0.321	0.663
rs3763763	TACC2	miR-92a-3p	Chr10: 122254089	C/A	252/196/40	276/200/36	0.266	0.977
rs8323	CX3CL1	miR-17-5p/miR-20a-5p	Chr16: 57384770	C/T	240/211/37	226/245/41	0.319	0.023

^a^Major/minor alleles; ^b^Numbers of major homozygote/heterozygote/minor homozygote; ^c^Minor allele frequency (MAF) in our controls; ^d^Hardy-Weinberg equilibrium (HWE) in our controls

**Table 3:** Associations of the selected SNPs with the risk of ESCC in four genetic models

	Additive model		Dominant model		Recessive model		Codominant model^b^			
							het		hom	
SNPs	OR (95%CI)^a^	*P*^a^	OR (95%CI)^a^	*P*^a^	OR (95%CI)^a^	*P*^a^	OR (95%CI)^a^	*P*^a^	OR (95%CI)^a^	*P*^a^
rs12594531	0.930 (0.773–1.118)	0.438	0.909 (0.692–1.193)	0.491	0.909 (0.650–1.269)	0.574	0.924 (0.694–1.231)	0.591	0.866 (0.594–1.263)	0.455
rs1366600	0.813 (0.638–1.036)	0.094	0.785 (0.594–1.037)	0.088	0.788 (0.367–1.692)	0.541	0.790 (0.592–1.055)	0.110	0.737 (0.342–1.591)	0.437
rs1804506	**0.817 (0.681–0.981)**	**0.030**	0.765 (0.578–1.012)	0.060	0.767 (0.559–1.054)	0.102	0.807 (0.600–1.085)	0.155	**0.670 (0.463–0.968)**	**0.033**
rs3741779	0.896 (0.736–1.092)	0.278	0.944 (0.732–1.218)	0.660	0.680 (0.432–1.069)	0.095	1.008 (0.772–1.316)	0.951	0.683 (0.426–1.093)	0.112
rs3763763	1.053 (0.860–1.288)	0.618	1.056 (0.818–1.363)	0.677	1.104 (0.682–1.786)	0.687	1.043 (0.798–1.363)	0.757	1.124 (0.685–1.845)	0.643

^a^adjusted for age, gender, smoking status and family history of cancers in logistic regression model

^b^*het* Heterozygote vs major homozygote, *hom* Minor homozygote vs major homozygote, *CI* Confidence interval, *OR* Odds ratio, *SNP* Single nucleotide polymorphism

Based on the demographic characteristics (age, gender, smoking status and family history of cancers), we further conducted stratification analysis to assess the associations of selected SNPs with ESCC risk under an additive model. As shown in Supplementary Table S3 (Additional file 2), the rs1804506 T allele showed more striking effects on reducing ESCC risk among older individuals (OR = 0.791, 95% CI = 0.638–0.982, *P* = 0.034), women (OR = 0.711, 95% CI = 0.524–0.966, *P* = 0.029) or non-smokers (OR = 0.747, 95% CI = 0.594–0.938, *P* = 0.012). For rs1366600, subjects carrying the G allele had a decreased susceptibility of ESCC in smokers (OR = 0.672, 95% CI = 0.456–0.991, *P* = 0.045) or individuals without family history of cancers (OR = 0.699, 95% CI = 0.501–0.976, *P* = 0.035).

### SNP-SNP and SNP-smoking interactions analysis

Further investigations of SNP-SNP and SNP-smoking interactions on the risk of ESCC were tested using the GMDR methods. After adjustment for age, gender, smoking and family history of cancers, the four-way interaction model (rs1366600, rs1804506, rs3741779 and rs3763763) was regarded as the optimal SNP-SNP model, which scored 9/10 for cross-validation consistency and 9 for sign test (*P* = 0.011) (Table 4).

**Table 4:** GMDR analysis for the best SNP-SNP and SNP-smoking interaction models

No. of loci	Best combination	Te-BA	Sign test (*P*)	CVC
SNP-SNP interaction^a^				
2	rs12594531, rs1366600	0.512	7 (0.172)	6/10
3	rs1804506, rs3741779, rs3763763	0.538	8 (0.055)	8/10
4	**rs1366600, rs1804506, rs3741779, rs3763763**	**0.558**	**9 (0.011)**	**9/10**
5	rs12594531, rs1366600, rs1804506, rs3741779, rs3763763	0.538	7 (0.172)	10/10
SNP-smoking interaction^b^				
2	**smoking, rs1366600**	**0.574**	**9 (0.011)**	**8/10**
3	smoking, rs12594531, rs1366600	0.525	7 (0.172)	4/10
4	smoking, rs12594531, rs1366600, rs1804506	0.538	9 (0.011)	5/10
5	smoking, rs1366600, rs1804506, rs3741779, rs3763763	0.526	6 (0.377)	4/10
6	smoking, rs12594531, rs1366600, rs1804506, rs3741779, rs3763763	0.552	8 (0.055)	10/10

^a^adjusted for age, gender, smoking status and family history of cancers

^b^adjusted for age, gender and family history of cancers

*GMDR* Generalized multifactor dimensionality reduction, *Te-BA* Testing-balanced accuracy, *CVC* Cross-validation consistency

In addition, the best SNP-smoking model for ESCC risk was found to be the interaction between rs1366600 and smoking (*P* = 0.011), which scored 8/10 for cross-validation consistency and 0.574 for testing accuracy from the GMDR analysis (Table 4). The joint effect of rs1366600 genotype and smoking on ESCC was further validated by logistic regression analysis. After adjustment for age, gender and family history of cancers, we found that non-smoking status was linked with a significantly lower risk of ESCC irrespective of the genetic risk profiles. Moreover, non-smoking subjects who carried the rs1366600 AG or GG genotype have a remarkably decreased risk of ESCC (OR = 0.320, 95% CI = 0.195–0.527, *P* < 0.001), compared with those having rs1366600 AA genotypes and smoking history (Table 5).

**Table 5:** Jointed effects of rs1366600 and smoking status on ESCC risk validated by the logistic regression model

Smoking status	rs1366600	Controls	Cases	ORs (95% CI)^a^	*P*^a^
	A/G	n (%)	n (%)		
With smoking	AA	261 (51.0)	192 (39.4)	1.000	
	AG + GG	118 (23.0)	70 (14.3)	0.739 (0.467–1.168)	0.195
Without smoking	AA	86 (16.8)	161 (33.0)	0.350 (0.237–0.516)	**< 0.001**
	AG + GG	47 (9.2)	65 (13.3)	0.320 (0.195–0.527)	**< 0.001**

^a^ORs (95% CI) and *P*-values were obtained by the logistic regression analysis after adjustment for age, gender and family history of cancers, *ESCC* Esophageal squamous cell carcinoma, *OR* Odds ratio, *CI* Confidence interval

### Expression of miR-19a-3p, miR-19b-3p and target gene TGFBR3 in ESCC tissues

**Fig. 1:**
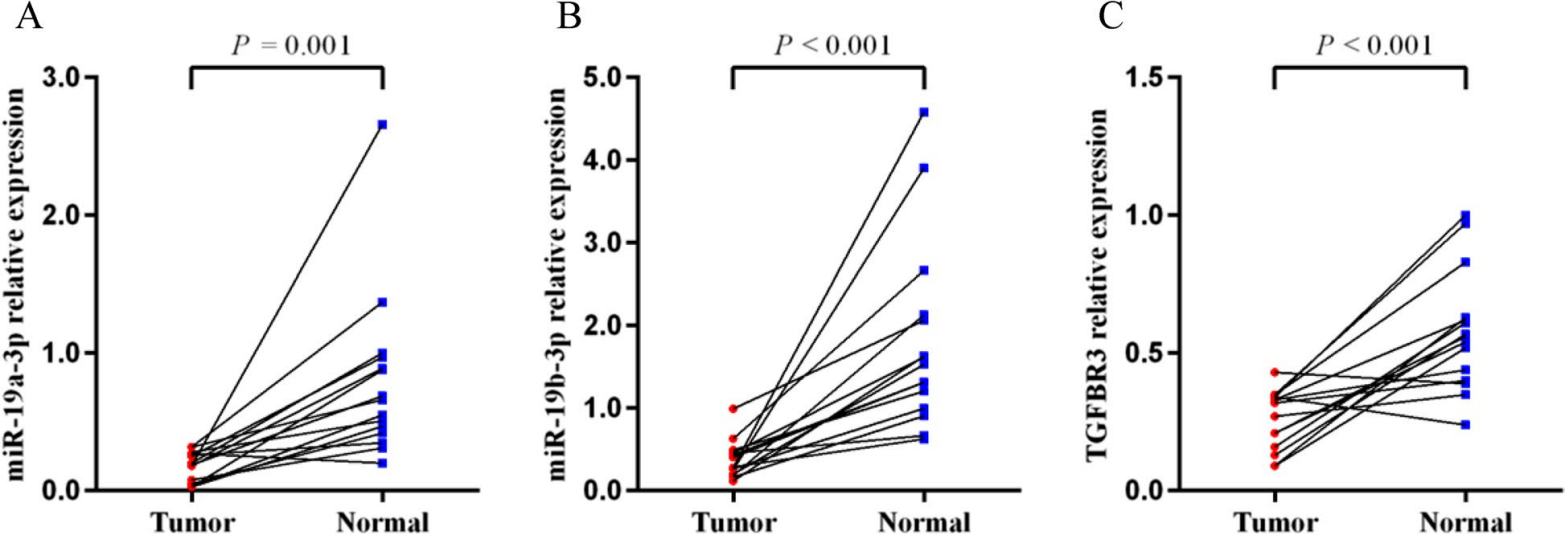
The expression of miR-19a-3p, miR-19b-3p and target gene TGFBR3 in ESCC tissues. **A** The expression of miR-19a-3p in ESCC tissues and adjacent normal tissues; **B** The expression of miR-19b-3p in ESCC tissues and adjacent normal tissues; **C** The expression of TGFBR3 in ESCC tissues and adjacent normal tissues

**Fig. 2:**
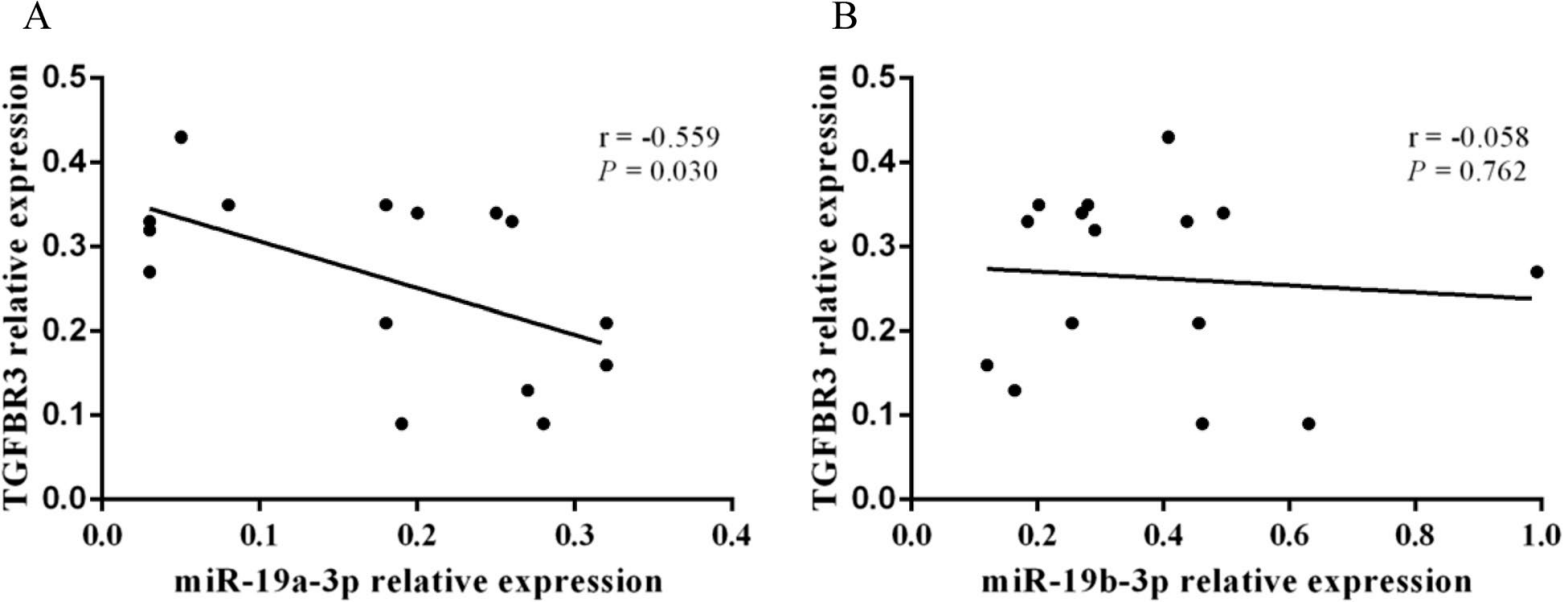
The correlations of miRNAs and target gene expression levels in colorectal cancer tissues. **A** Correlation analysis between miR-19a-3p and TGFBR3 expression levels in ESCC tissues; **B** Correlation analysis between miR-19b-3p and TGFBR3 expression levels in ESCC tissues

**Fig. 3:**
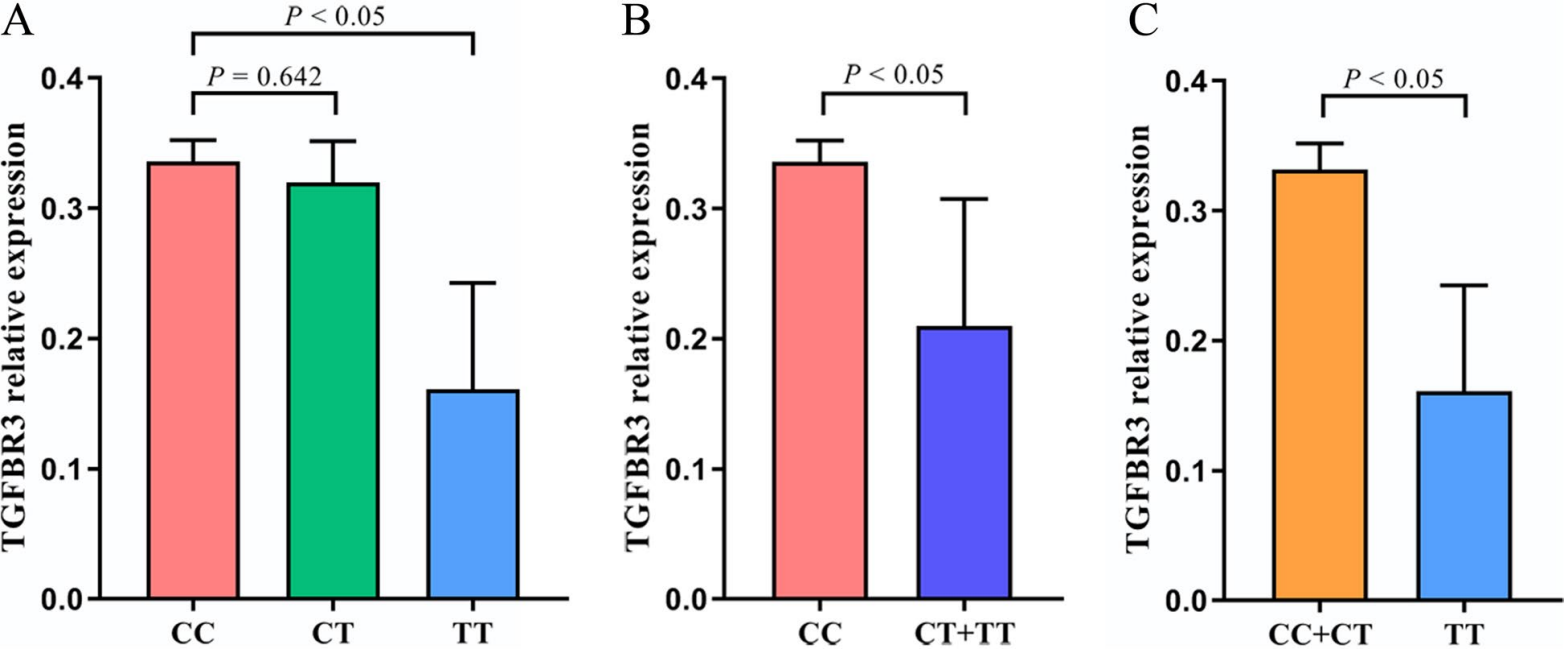
The expression level of TGFBR3 in ESCC tissues from patients with rs1804506 CC, CT and TT genotypes on miR-19b-3p: TGFBR3 binding. **A** Codominant model; **B** Dominant model; (C) Recessive model

Since SNP rs1804506 was significantly associated with ESCC in our Chinese Han population, the expression levels of its located gene (TGFBR3) and the interacted miRNAs (miR-19a-3p and miR-19b-3p) were next detected in ESCC tissues and adjacent normal tissues. The result showed that both miR-19a-3p and miR-19b-3p were significantly downregulated in ESCC tissues compared to normal tissues (*P* = 0.001 and *P* < 0.001, respectively) (Fig. 1A-B). The expression level of TGFBR3 was significantly lower in ESCC tissues than that in normal tissues (*P* < 0.001) (Fig. 1C). Besides, a negative correlation between miR-19a-3p and TGFBR3 expression was discovered in ESCC tissues (*r* = − 0.559, *P* = 0.030) (Fig. 2A), and no significant correlation was found between miR-19b-3p and TGFBR3 expression (*r* = − 0.058, *P* = 0.762) (Fig. 2B). Furthermore, we evaluated the expression level of TGFBR3 in ESCC patients with different genotypes, and found that individuals carrying rs1804506 TT genotype had significantly decreased TGFBR3 expression in cancerous tissues than those carrying rs1804506 CC genotype (Fig. 3A). As shown in Fig. 3, the expression level of TGFBR3 was significantly lower in cancerous tissues of rs1804506 T carriers (Fig. 3B), while the rs1804506 C carriers presented higher TGFBR3 expression in cancerous tissues (Fig. 3C).

### eQTL analysis of rs1804506

We further queried eQTL databases to explore the association between rs1804506 and TGFBR3 expression. Results of the eQTL analysis from the Multiple Tissue Human Expression Resource (MuTHER) project showed that there was a significant association between rs1804506 and TGFBR3 expression in subcutaneous adipose tissue (*P* = 3.44e-05). Additionally, we also searched the GTEx database and found no significant association between rs1804506 and TGFBR3 expression in esophageal-related tissues, whereas rs1804506 was identified as splicing quantitative trait locus (sQTL) at the esophagogastric junction (*P* = 1.10e-05). Moreover, eQTLGen, containing 31,684 blood samples, indicated that rs1804506 was a significant cis-eQTL of CDC7 (*P* = 8.47e-08).

### SNP rs1804506 interferes the binding between miR-19a-3p and TGFBR3 3′-UTR

**Fig. 4:**
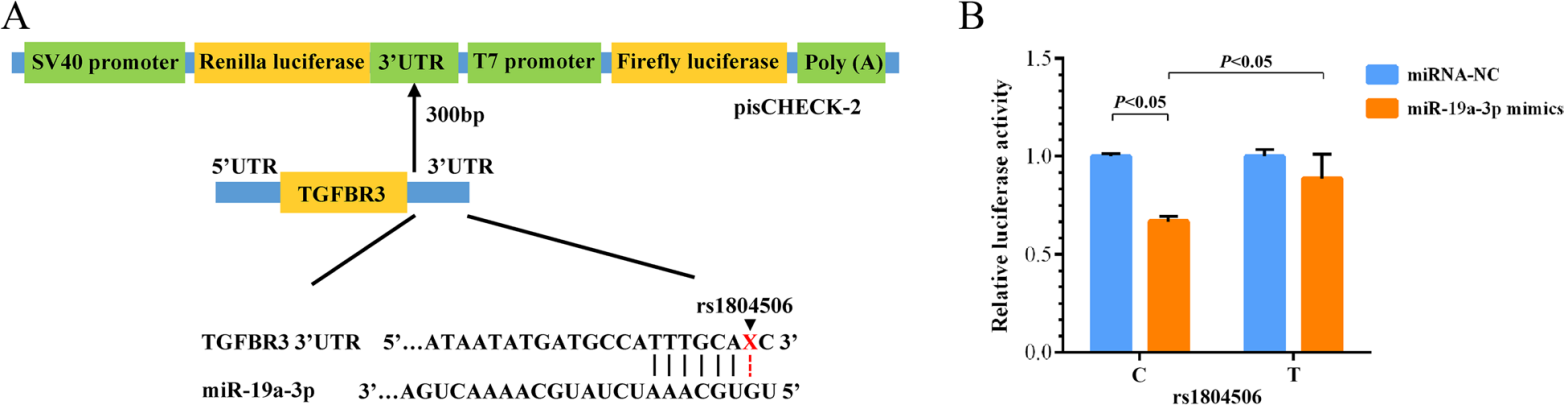
The effect of rs1804506 on the binding of miR-19a-3p and TGFBR3. **A** Schematic diagram of constructed plasmids containing the TGFBR3 3′-UTR and predicted binding sites between miR-19a-3p and the TGFBR3 3′-UTR; **B** Dual luciferase reporter assay was performed to measure the relative luciferase activity in the rs1804506-C plasmids and rs1804506-T plasmids cotransfected with has-miR-19a-3p mimics or negative control (NC) in HEK-293 T cells

In-silico analysis suggested that SNP rs1804506 located in the putative binding sites between miR-19a-3p and TGFBR3. To demonstrate whether rs1804506 altered the binding capacity of miR-19a-3p and TGFBR3, we performed dual-luciferase reporter assay by constructing plasmids containing TGFBR3 3′-UTR (Fig. 4A). The results showed that the luciferase activity significantly reduced in the rs1804506-C plasmids compared with the rs1804506-T plasmids in HEK-293 T cells (*P* < 0.05) (Fig. 4B). These finding suggested that miR-19a-3p might directly target the TGFBR3 3′-UTR with the rs1804506 C allele. SNP rs1804506 could affect the binding between miR-19a-3p and the TGFBR3 3′-UTR.

## Discussion

Genetic variants in miRNA targeting sites play important roles in cancer development and might contribute to the risk of different types of cancer. The miR-17-92 cluster is involved in the occurrence and development of various cancers. Recent studies have focused on the association between polymorphisms in the binding sites of miR-17-92 cluster and cancer susceptibility. SNP rs1053667 located in the 3’UTR binding region of miR-19b-3p and KIAA0423 was closely related to the survival of esophageal adenocarcinoma [30]. Another case-control study has demonstrated that the C-C-G-C-A haplotype comprised of five SNPs (rs1805110, rs2810904, rs1805112, rs284878, and rs1804506) was associated with the risk of hepatocellular carcinoma [31]. However, to the best of our knowledge, SNPs in binding sites of miR-17-92 cluster have not been investigated in ESCC. In this study, we evaluated the association between the miR-SNPs located in the binding sites of miR-17-92 cluster and ESCC by a case-control study design and explored the putative function of candidate miR-SNPs with molecular biology experiments. Our results showed that rs1804506 TT genotype was significantly associated with a reduced risk of ESCC. The rs1804506 T allele could decrease the risk of ESCC in the older, female or non-smoker subgroups. Moreover, we found that the rs1804506 was correlated to the expression level of TGFBR3, which has not been reported so far.

Accumulating evidence suggests that ESCC is a complex disease, and it is affected by multiple genetic and environmental factors. According to our results, after adjusting for age, gender, smoking status and family history of cancers, a four-way interaction was observed among rs1366600, rs1804506, rs3741779, and rs3763763. Besides, we found that the miR-17-92 cluster related SNP rs1366600 interacted with smoking and contributed to ESCC risk, which has not been reported so far. With regards to rs1804506, the stratified analysis results showed that rs1804506 T allele obviously decreased the susceptibility of ESCC in non-smokers. Our findings indicated that non-smokers with rs1366600 AG/GG genotype had much lower ESCC risk compared to the smokers with rs1366600 AA genotype. For individuals at a low genetic risk of rs1366600 AG or GG genotype, they were more likely to benefit from quitting smoking, thus lowering their risk of developing ESCC.

Several studies have discovered the interaction between smoking and miRNA-related genetic variants in the development of ESCC. Our previous findings suggested that miR-15b related SNP rs1451761 interacted with smoking to reduce the risk of ESCC [14]. In addition, the BRCA1 rs799917 polymorphism was reported to interact with tobacco, which is associated with the intensified susceptibility to ESCC [32]. It could be explained by the evidence that the high level of benzo(a) pyrene (BaP) in tobacco smoke activated the upregulation of miR-638, leading to a decreased expression of BRCA1 in esophageal epithelia cells with rs799917 C allele [33]. Nevertheless, more attention is needed to clarify the interaction effect between smoking and miR-17-92 cluster related SNPs in ESCC.

The miR-17-92 cluster has been well studied and been considered as a key mediator in the development of a variety of malignant tumors. As members of the miR-17-92 cluster, miR-19a and miR-19b have been shown to be differentially expressed in various cancers [21–23]. They were also reported to be highly expressed in ESCC tissues, and miR-19a could serve as a potential prognostic biomarker for ESCC [22, 34]. However, the expression of miR-19a and miR-19b in ESCC remains controversial. In the present study, we observed that miR-19a-3p and miR-19b-3p were down-regulated in ESCC tissues compared with the adjacent normal tissues. Additionally, we also found significantly decreased expression of miR-19a and miR-19b in ESCC tissues by the GEO2R analysis from GEO datasets (data was not shown). Our conflicting results with the previous study might result from the different populations in these studies, which needed further validation in other independent cohorts. TGFBR3, an essential co-receptor of the TGF-β superfamily, is involved in cancer cell migration, invasion and metastasis, and exerts tumor-suppressive roles in various cancers [35]. Although one recent study has reported that the high level of TGFBR3 protein was correlated with TNM staging of ESCC [36], the dysregulation of TGFBR3 in ESCC has not been identified at the mRNA level. Our results showed that TGFBR3 was significantly downregulated in ESCC tissues compared with adjacent normal tissues, which indicated that TGFBR3 might be involved in the development of ESCC.

In the present study, we found a negative correlation between the expression of miR-19a-3p and TGFBR3 in ESCC tissues (*r* = − 0.559, *P* = 0.030), whereas no significant association was discovered in normal tissues (*r* = 0.326, *P* = 0.235). We also assessed the expression levels of TGFBR3 in the tissues from ESCC patients with different genotypes of rs1804506, and the rs1804506 T allele appears to be associated with decreased expression of TGFBR3. According to the GTEx database, rs1804506 was located in sQTL, which might also affect alternative splicing of TGFBR3 at the esophagogastric junction (*P* = 1.10e-05). The occurrence of alternative splicing could alter gene expression and is involved in tumorigenesis and development [37, 38]. Frameshifts induced by alternative splicing could cause the nonsense mediated mRNA decay and the down-regulation of gene expression [39], which might explain the decreased expression of TGFBR3 in the rs1804506 T allele carriers. In addition, eQTL analysis results suggested that rs1804506 was significantly associated with the expression level of TGFBR3 in subcutaneous adipose tissue (*P* = 3.44e-05). However, the majority of the samples in the GTEx database came from European American (85.3%) and African American populations (12.3%) [29], which were quite different from the Chinese population in terms of incidence, genetic backgrounds, and susceptibility to esophageal cancer. Therefore, future studies are needed to investigate the underlying relationship and mechanism between rs1804506 and TGFBR3 expression.

It has been noticed that miR-SNPs in binding sites could affect cancer susceptibility through affecting the binding ability and then modifying the expression levels of targeted genes. The SNP rs16917496 could effectively change the binding affinity between miR-502 and SET8 and downregulate SET8 expression, which was associated with the prognosis of ESCC [40]. The SNP rs2866943 located in PTPRT 3′-UTR was reported to disrupt the regulation of miR-218 on the PTPRT expression and played a protective role in ESCC pathogenesis [41]. The mutation of rs1595066 located in ERBB4 generated a new binding site with miR-200 and might reduce ESCC risk by suppressing ERBB4 expression [42]. In the present study, we found that the luciferase activity was significantly lower in the rs1804506-C plasmids compared with the rs1804506-T plasmids, which revealed that SNP rs1804506 C > T might affect the binding capacity between miR-19a-3p and the TGFBR3 3′-UTR. As a consequence, we speculated that rs1804506 might affect the post-transcriptional regulation of miR-19a-3p by changing the binding ability of miR-19a-3p and TGFBR3, thus altered the expression level of TGFBR3 and contributed to ESCC risk. Nevertheless, the biological roles and mechanism of rs1804506 in ESCC still needs to be further elucidated by in vivo and in vitro experiments.

Some limitations should be taken into account in this study. First of all, the genotype of candidate SNPs were detected based on a relatively small sample size, and the case and control groups failed to be matched by age and sex, which could result in selection bias. However, we performed multivariable analysis and stratified analysis to reduce the potential bias and confounding. Secondly, the expression levels of miR-19a-3p and TGFBR3 were measured in a limited number of ESCC patients. The expression pattern of miRNA and target genes as well as their association with miR-SNPs still need further validation in a large population. Finally, despite the association between the mutation of rs1804506 polymorphism and TGFBR3 expression, more functional experiments are urgent to clarify the specific biological functions of rs1804506 in the regulation of miR-19a-3p and TGFBR3 in future studies.

## Conclusions

In summary, this study investigated the relationship between the polymorphisms and expression of miR-17-92 cluster and ESCC risk based on a population-based case-control study design. The SNP rs1804506 was associated with the decreased risk of ESCC. The interaction effect between rs1366600 and smoking might play a role in the pathogenesis of ESCC. Moreover, the rs1804506 C > T variant was related to the expression level of TGFBR3 and might change the binding activity of miR-19a-3p and TGFBR3 3’UTR. Our study provided new evidence that SNP rs1804506 in miR-17-92 cluster binding sites might serve as a functional susceptibility locus and a promising biomarker for the prevention and treatment of ESCC. However, further studies in large populations and more functional investigations are warranted to validate the findings in this study.

## Supplementary Information

**Additional file 1: Supplementary Figure S1.** The predicted miRNA binding sites of the studied miR-SNPs.

**Additional file 2: Supplementary Table S1.** Primers sequences used in this study. **Supplementary Table S2.** Clinical characteristics of ESCC tissues. ESCC, esophageal squamous cell carcinoma; SD, standard deviation. **Supplementary Table S3.** Stratified analysis of the five selected SNPs and ESCC susceptibility^a^.

## Data Availability

The datasets generated or analysed during this study are included in this article and its additional files.

## Abbreviations

EC: Esophageal cancer
ESCC: Esophageal squamous cell carcinoma
SNP: Single nucleotide polymorphisms
miRNA: microRNA
3′-UTR: 3′-untranslated region
mRNA: Messenger RNA
miR-SNP: miRNA-related single nucleotide polymorphism
ESD: Esophageal squamous dysplasia
MAF: Minor allele frequency
eQTL: Expression quantitative trait loci
OR: Odds ratio
CI: Confidence interval
GMDR: Generalized multifactor dimensionality reduction
sQTL: Splicing quantitative trait locus
