# Supplementary material for: Genetic variant in miR-17-92 cluster binding sites is associated with esophageal squamous cell carcinoma risk in Chinese population
Source: BMC Cancer. 2022 Dec 2;22:1253. doi: 10.1186/s12885-022-10360-6 (PMC9719157; doi:10.1186/s12885-022-10360-6)
Supplement: Supplementary file 1 — Additional file 1: Supplementary Figure S1. The predicted miRNA binding sites of the studied miR-SNPs. [file 12885_2022_10360_MOESM1_ESM.pdf]

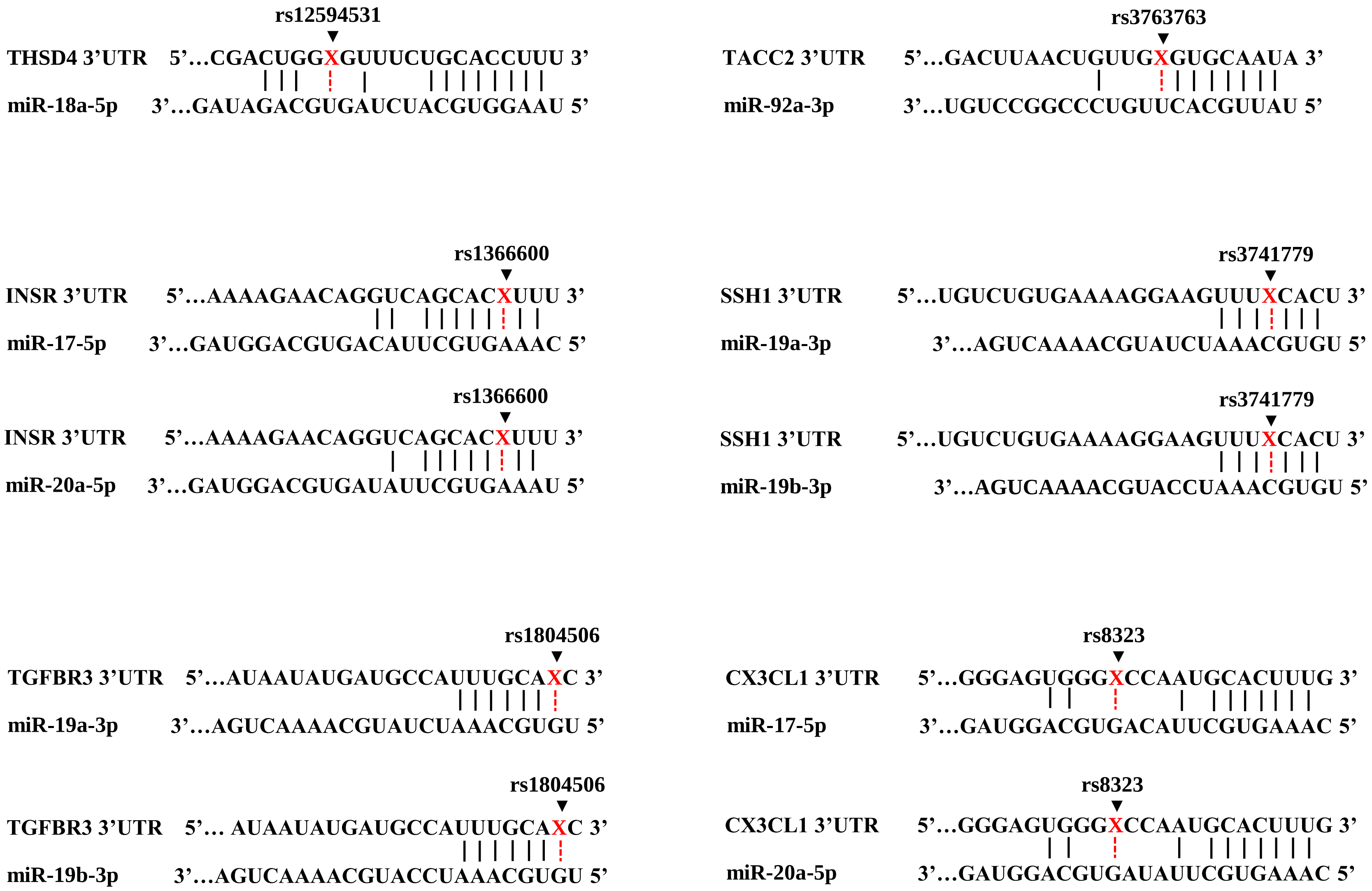

Supplementary Fig. S1 The predicted miRNA binding sites of the studied miR-SNPs
