# Supplementary material for: Genetic variant in miR-17-92 cluster binding sites is associated with esophageal squamous cell carcinoma risk in Chinese population
Source: BMC Cancer. 2022 Dec 2;22:1253. doi: 10.1186/s12885-022-10360-6 (PMC9719157; doi:10.1186/s12885-022-10360-6)
Supplement: Supplementary file 2 — Additional file 2: Supplementary Table S1. Primers sequences used in this study. Supplementary Table S2. Clinical characteristics of ESCC tissues. ESCC, esophageal squamous cell carcinoma; SD, standard deviation. Supplementary Table S3. Stratified analysis of the five selected SNPs and ESCC susceptibilitya. [file 12885_2022_10360_MOESM2_ESM.docx]

Supplementary Table S1 Primers sequences used in this study

| Description | Forward primer sequence (5’-3’) | Reverse primer sequence (5’-3’) |
| --- | --- | --- |
| MassARRAY for genotyping |  |  |
| rs12594531 | ACGTTGGATGGCCTAGGACCCTATAGATAC | ACGTTGGATGAGGGAGTTACTTTGCCCCTG |
| rs1366600 | ACGTTGGATGTATATGCGAGGTGAGGTTGG | ACGTTGGATGCCTGAAAGTCCACCATGTTC |
| rs1804506 | ACGTTGGATGACAAGGAGGTATCACTGAGC | ACGTTGGATGAAGGGCTTGAGGTGAATTTC |
| rs3741779 | ACGTTGGATGGCAAAGACGAATTCATGCTG | ACGTTGGATGAAAGCAAGGGCCAAGCTATG |
| rs3763763 | ACGTTGGATGACAGAACCTGTCCATGGAAC | ACGTTGGATGCTGAACCGAATGTTTTGGAC |
| rs8323 | ACGTTGGATGCATATTCAGGAAGCTCAGGG | ACGTTGGATGGGAAGAGCCAGAACAAAGTG |
| RT |  |  |
| U6 | CGCTTCACGAATTTGCGTGTCAT |  |
| miR-19a-3p | GTCGTATCCAGTGCGTGTCGTGGAGTCGGCAATTGCACTGGATACGACTCAGTTTTG | |
| miR-19b-3p | GTCGTATCCAGTGCGTGTCGTGGAGTCGGCAATTGCACTGGATACGACTCAGTTTTG | |
| qRT-PCR |  |  |
| U6 | GCTTCGGCAGCACATATACTAAAAT | CGCTTCACGAATTTGCGTGTCAT |
| β-actin | GTGGCCGAGGACTTTGATTG | CCTGTAACAACGCATCTCATATT |
| miR-19a-3p | GGGGGGGTGTGCAAATCT | GTGCGTGTCGTGGAGTCG |
| miR-19b-3p | GGGGGGTGTGCAAATCC | GTGCGTGTCGTGGAGTCG |
| TGFBR3 | AAGTGACTGGACGAGACG | GCAATCACATAATGGGAAG |

Supplementary Table S2 Clinical characteristics of ESCC tissues

| Variables | N=15 |
| --- | --- |
| Age (years, mean ± SD) | 65.27 ± 7.61 |
| Gender |  |
| Male | 8 |
| Female | 7 |
| Tumor location |  |
| Upper | 2 |
| Middle | 12 |
| Lower | 1 |
| Differentiation |  |
| Poor | 2 |
| Medium | 11 |
| High | 2 |
| TNM stage |  |
| І+Ⅱ | 5 |
| Ⅲ+Ⅳ | 10 |

ESCC, esophageal squamous cell carcinoma; SD, standard deviation.

Supplementary Table S3 Stratified analysis of the five selected SNPs and ESCC susceptibility^a^

| Variables | rs12594531 | |  | rs1366600 | |  | rs1804506 | |  | rs3741779 | |  | rs3763763 | |
| --- | --- | --- | --- | --- | --- | --- | --- | --- | --- | --- | --- | --- | --- | --- |
|  | OR (95% CI) | *P* |  | OR (95% CI) | *P* |  | OR (95% CI) | *P* |  | OR (95% CI) | *P* |  | OR (95% CI) | *P* |
| Age |  |  |  |  |  |  |  |  |  |  |  |  |  |  |
| ≤60 | 0.804(0.554-1.167) | 0.251 |  | 0.639(0.407-1.003) | 0.052 |  | 0.918(0.647-1.304) | 0.633 |  | 0.796(0.550-1.152) | 0.226 |  | 1.277(0.867-1.879) | 0.216 |
| >60 | 0.956(0.772-1.185) | 0.683 |  | 0.892(0.667-1.192) | 0.440 |  | **0.791(0.638-0.982)** | **0.034** |  | 0.916(0.722-1.161) | 0.469 |  | 0.947(0.744-1.204) | 0.656 |
| Gender |  |  |  |  |  |  |  |  |  |  |  |  |  |  |
| Female | 0.988(0.724-1.349) | 0.942 |  | 0.808(0.524-1.246) | 0.334 |  | **0.711(0.524-0.966)** | **0.029** |  | 0.743(0.532-1.037) | 0.081 |  | 0.976(0.683-1.394) | 0.894 |
| Male | 0.890(0.706-1.121) | 0.322 |  | 0.805(0.598-1.083) | 0.152 |  | 0.906(0.719-1.142) | 0.402 |  | 0.973(0.757-1.251) | 0.833 |  | 1.041(0.811-1.336) | 0.754 |
| Smoking status | |  |  |  |  |  |  |  |  |  |  |  |  |  |
| Never | 0.908(0.721-1.144) | 0.414 |  | 0.912(0.670-1.243) | 0.561 |  | **0.747(0.594-0.938)** | **0.012** |  | 0.854(0.670-1.088) | 0.201 |  | 1.047(0.810-1.353) | 0.728 |
| Current /former | 0.982(0.722-1.334) | 0.905 |  | **0.672(0.456-0.991)** | **0.045** |  | 0.994(0.728-1.357) | 0.969 |  | 0.996(0.703-1.411) | 0.981 |  | 1.032(0.742-1.436) | 0.85 |
| Family history of cancers | |  |  |  |  |  |  |  |  |  |  |  |  |  |
| No | 1.044(0.816-1.336) | 0.730 |  | **0.699(0.501-0.976)** | **0.035** |  | 0.860(0.672-1.100) | 0.230 |  | 0.928(0.713-1.207) | 0.577 |  | 1.039(0.794-1.359) | 0.78 |
| Yes | 0.804(0.608-1.063) | 0.126 |  | 0.970(0.678-1.386) | 0.866 |  | 0.771(0.586-1.012) | 0.061 |  | 0.864(0.640-1.167) | 0.341 |  | 1.071(0.786-1.458) | 0.665 |

OR with 95% CI and *P*-value for adjusted logistic regression analysis under additive model; ESCC, esophageal squamous cell carcinoma; SNP, single-nucleotide polymorphism; OR, odds ratio; CI, confidence interval.
